# Ultra-sensitive all-fibre photothermal spectroscopy with large dynamic range

**DOI:** 10.1038/ncomms7767

**Published:** 2015-04-13

**Authors:** Wei Jin, Yingchun Cao, Fan Yang, Hoi Lut Ho

**Affiliations:** 1 Department of Electrical Engineering and Photonics Research Center, The Hong Kong Polytechnic University, Hung Hom, Kowloon, Hong Kong, China; 2 Photonic Sensors Research Center, The Hong Kong Polytechnic University Shenzhen Research Institute, No. 18 Yuexing 1st Road, Nanshan District, Shenzhen 518057, China

## Abstract

Photothermal interferometry is an ultra-sensitive spectroscopic means for trace chemical detection in gas- and liquid-phase materials. Previous photothermal interferometry systems used free-space optics and have limitations in efficiency of light–matter interaction, size and optical alignment, and integration into photonic circuits. Here we exploit photothermal-induced phase change in a gas-filled hollow-core photonic bandgap fibre, and demonstrate an all-fibre acetylene gas sensor with a noise equivalent concentration of 2 p.p.b. (2.3 × 10^−9^ cm^−1^ in absorption coefficient) and an unprecedented dynamic range of nearly six orders of magnitude. The realization of photothermal interferometry with low-cost near infrared semiconductor lasers and fibre-based technology allows a class of optical sensors with compact size, ultra sensitivity and selectivity, applicability to harsh environment, and capability for remote and multiplexed multi-point detection and distributed sensing.

## Abstract (web summary)

Photothermal interferometry systems using free-space optics have limits in terms of light–matter interaction efficiency, size, optical alignment and integration. Here, Jin *et al*. use a gas-filled hollow-core photonic bandgap fibre to demonstrate an all-fibre gas sensor with ultrahigh sensitivity and dynamic range.

## Graphical abstract

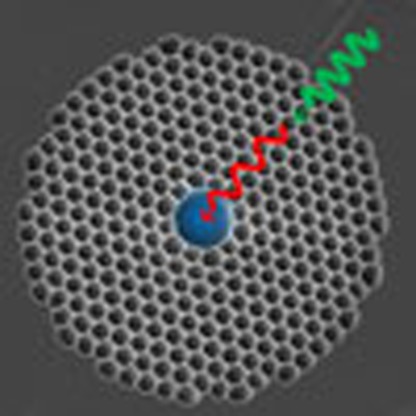

Detection of trace chemicals sensitively and selectively is important for environmental, safety and industrial process monitoring as well as national security applications. Laser absorption spectroscopy (LAS), which relies on the ‘finger-print' absorption lines of molecules for identifying and detecting trace chemicals, is a powerful technique that offers high selectivity and sensitivity^1^,^2^,^3^,^4^. The employment of optical fibre-based technologies greatly expands the capability of LAS for remote interrogation, space limited and harsh environmental applications, multiplexed multi-point detection, and sensor networking^3^,^4^,^5^,^6^. However, fibre-based LAS sensors have limitations in performances. Conventional silica optical fibres have a low-loss transmission window from ∼0.5 to ∼1.8 μm and only allow access to the relatively weak overtone absorption lines of molecules, which significantly compromises the detection sensitivity. Taking gas detection as an example, fibre-pigtailed open-path and evanescent wave gas cells have demonstrated noise equivalent concentration (NEC) of 10–100 p.p.m.^3^,^4^,^5^,^6^, with a linear dynamic range of two to three orders of magnitude^7^. Hollow-core photonic bandgap fibre (HC-PBF) gas cells enable stronger light–gas interaction over a longer distance and have demonstrated a NEC of ∼10 p.p.m. methane with 5.1-m-long HC-PBF cells^8^, corresponding to a noise equivalent absorption coefficient (NEA) of 1.6 × 10^−6^ cm^−1^. Further significant increase in detection sensitivity is challenging since it requires an impractically long length of HC-PBF, which also compromises the response time of the sensor significantly.

Instead of direct measurement of spectral attenuation, here we exploit photothermal (PT) effect in a HC-PBF and demonstrate ultra-sensitive gas detection by probing the gas-absorption-induced phase change via fibre-optic interferometry. Optical absorption of molecules results in localized heating, which modifies the temperature, pressure and density, and modulates the refractive index (RI) of the material^9^. The RI modulation can be probed by a range of techniques such as PT lens^10^, deflection^11^, diffraction^12^ and interferometry^13^,^14^. PT interferometry measures the change of optical phase accumulated over a propagation distance and has been demonstrated for detecting weak absorbance in gas- and liquid-phase materials with very high sensitivity^13^,^14^,^15^,^16^,^17^. However, all the PT interferometry systems reported previously use free-space optics with an absorption distance up to several tens of centimetres, limited by beam divergence, optical loss and size of set-up. To access the stronger absorption lines, high-power gas lasers operating at the mid-infrared (MIR) were typically employed and they are complex, bulky and incompatible with fibre-optic systems.

In this article, we report an all-fibre PT interferometry system that uses a HC-PBF operating at the near infrared (NIR) wavelengths and demonstrate remarkably sensitive trace-gas detection with an unprecedented dynamic range. The HC-PBF confines the fluidic sample and propagating light modes simultaneously within the hollow-core, and the overlaps between the sample and the light fields approach 100% (refs 18, 19, 20). Light focused in a HC-PBF is much tighter and the interaction length is much longer, and the overall efficiency of light–sample interaction could be made significantly higher than a free-space PT system. This enables extremely high performance trace chemical detection systems operating at the NIR wavelengths, which are fully compatible with the telecom optical fibre technologies.

## Results

### Theory of PT spectroscopy in HC-PBF

**Figure 1:**
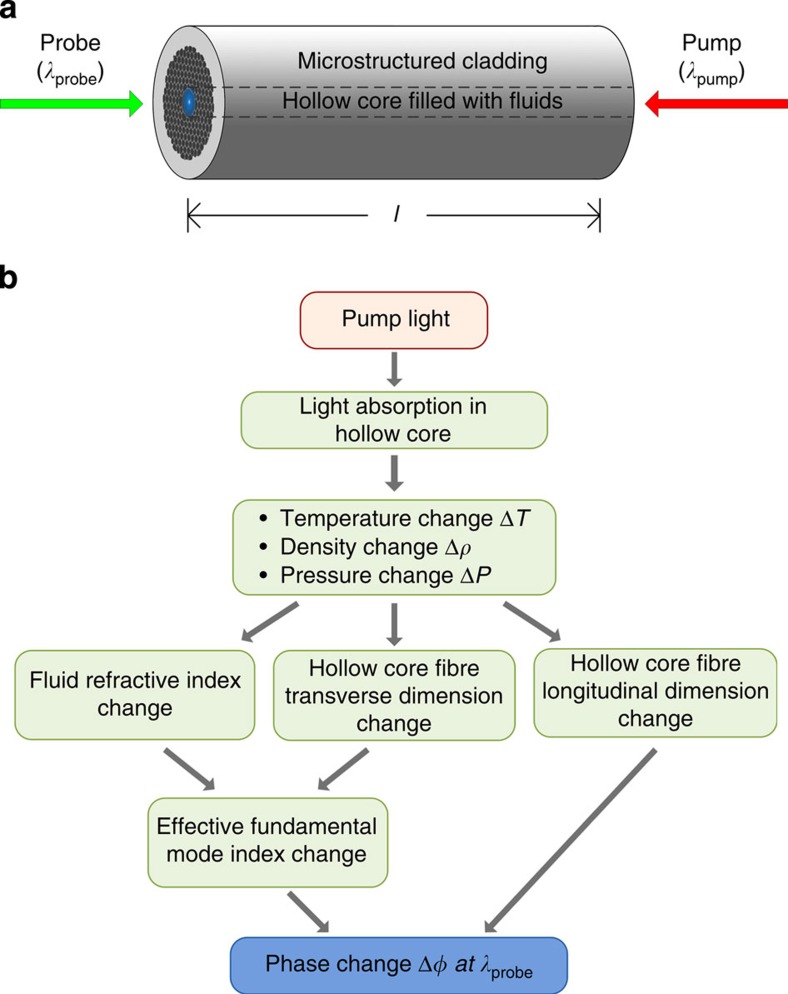
PT-induced phase modulation in a HC-PBF. (**a**) Modulated pump beam (*λ*_pump_) and constant probe beam (*λ*_probe_) are counter-propagating in a fluid-filled HC-PBF. The pump and probe may also be arranged to co-propagate within the HC-PBF. (**b**) Processes involved in producing phase modulation in a HC-PBF.

The basics of PT spectroscopy in a HC-PBF may be explained with the aid of Fig. 1. When a wavelength/intensity modulated pump light beam is coupled into a fluid-filled HC-PBF, it interacts with the spectrally absorbing fluid; the fluid molecules are excited to higher energy states and then return to their initial state via molecular collision. This process is accompanied by periodic heat production, which modulates the local temperature, density and pressure, and hence the RI of the fluid. The temperature and pressure modulation also perturb the transverse and longitudinal dimensions of the HC-PBF^21^,^22^. When a probe light beam is propagating along the same HC-PBF, the accumulated phase of the fundamental optical mode of the probe will be modulated.

Taking trace-gas detection as an example, for a pump beam propagating in a HC-PBF filled with a weakly absorbing gas, the intensity profile *I(r*, *z)* of the fundamental mode may be approximated by a Gaussian distribution^23^


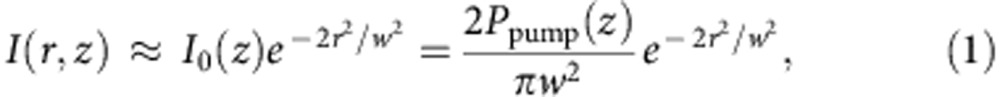


where *I*_0_(*z*) is the pump light intensity at the centre of the fibre core, which decreases with propagation distance *z* due to gas absorption and intrinsic fibre attenuation. *P*_pump_ is the pump power and 2*w* the mode field diameter (MFD), which has a value similar to the diameter of the hollow core. Assuming the heat yield is *Y_H_*, the local heat production rate due to weak optical absorption may be expressed as^9^


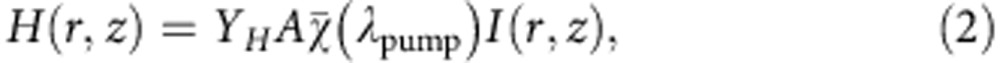


where 
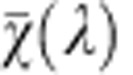
 is the peak normalized absorption line-shape function. *A*=*αC* is the peak absorption coefficient, *C* the relative gas concentration and *α* the peak absorption coefficient for a relative concentration of 100%. The heating process changes the temperature, density and pressure distribution of the fluid within the hollow core. The central portion of gas sample within the HC-PBF is heated up more, which reduces the density in the centre resulting in lower RI. This modulates the effective RI (*n*_eff_) of the fundamental mode as well as the length (*l*) of the HC-PBF. To a first order approximation, the fractional change in effective RI of the fundamental mode (*η*=Δ*η*_eff_/*η*_eff_) and length (*ɛ*=Δ*l*/*l*) at location *z* along the fibre may be expressed as


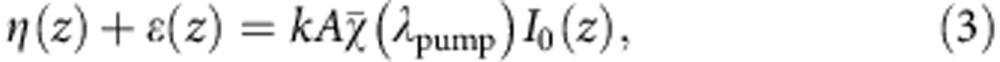


where *k* is a proportionality coefficient. The accumulated phase change *dφ* of the fundamental mode over interval [*z*, *z*+*dz*] at the probe wavelength (*λ*_probe_) may then be expressed as


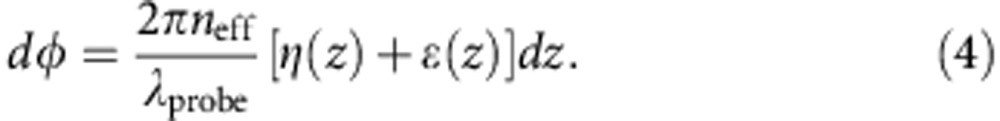


The phase change Δ*φ* over a propagation distance *L* may be obtained by integrating equation (4) and using equations (1) and (3), as


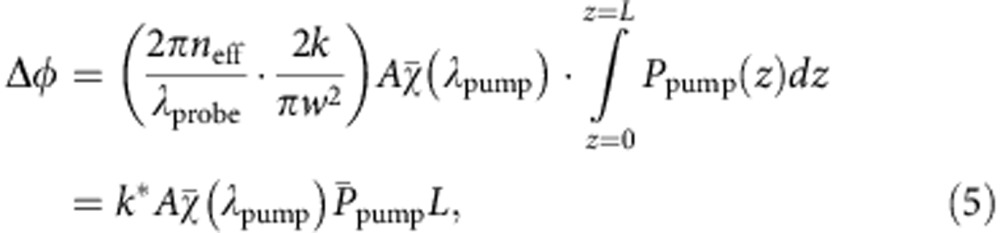


where 
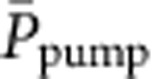
 represents the average pump power over length *L* which, for weak absorption and short fibre length, equals approximately the pump power inputting into the HC-PBF. k* is a coefficient proportional to 1/*w*^2^. The phase modulation may be recovered accurately by use of highly sensitive fibre-optic interferometry.

### Experimental set-up

**Figure 2:**
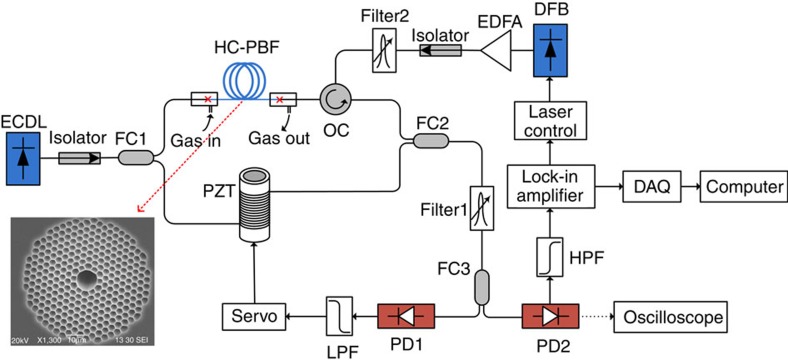
Experimental set-up for gas detection with 10-m-long HC-PBF. The splitting ratios of FC1 and FC2 are, respectively, 80/20 and 50/50, which approximately balanced the powers in the two arms of the interferometer. The optical path lengths of the two arms are also balanced to minimize the laser phase to intensity noise conversation. Filter 1 is used to filter out the residual pump and Filter 2 to minimize the effect of EDFA's ASE noise. The splitting ratio of FC3 is 90/10. Output from PD1 passes a low-pass-filter (LPF) and is used for interferometer stabilization. Output from PD2 contains the PT-induced phase modulation signal. The driving current of the DFB is modulated at 50 kHz by use of the internal signal generator of the lock-in. Inset: scanning electron microscopy (SEM) image of the HC-1550-02 fibre's cross-section. DAQ, data acquisition; DFB, distributed feedback laser (the pump); ECDL, external-cavity diode laser (the probe); EDFA, erbium-doped fibre amplifier; FC1-FC3, fibre couplers; OC, optical circulator; PD1-PD2, photo-detectors; PZT, piezoelectric transducer.

The basic experimental set-up for the study of PT-induced phase modulation is shown in Fig. 2. The sensing HC-PBF is the NKT Photonics' HC-1550-02 fibre with hollow core diameter of ∼11 μm. A distributed feedback semiconductor pump laser is modulated at 50 kHz and its wavelength is tuned to the P(9) line of acetylene (C_2_H_2_) at 1,530.371 nm. The acetylene molecular line strength at this wavelength is 1.211 × 10^−20^ cm per molecule, corresponding to line strength of 0.3 cm^−2^ atm^−1^ at room temperature^24^. The peak absorption coefficient of the line for a relative concentration of 100% at room temperature (296 K) and 1 atm is then calculated to be *α*=1.165 cm^−1^. The HC-PBF forms the sensing arm of a fibre-optic Mach-Zehnder interferometer (MZI) while the reference arm is made of a standard single-mode fibre (SMF) coiled around a piezoelectric transducer (PZT). The PZT acts as a phase compensator to keep the MZI at quadrature via a servo-loop^25^ (see Supplementary Fig. 1 and Supplementary Discussion). The MZI is probed by an external-cavity diode-laser with its wavelength tuned to 1,556.59 nm where the absorption of acetylene is minimal. The MZI output (PD2) is demodulated by use of a lock-in amplifier and the output waveform can also be observed from an oscilloscope.

### Test of lower detection limit

**Figure 3:**
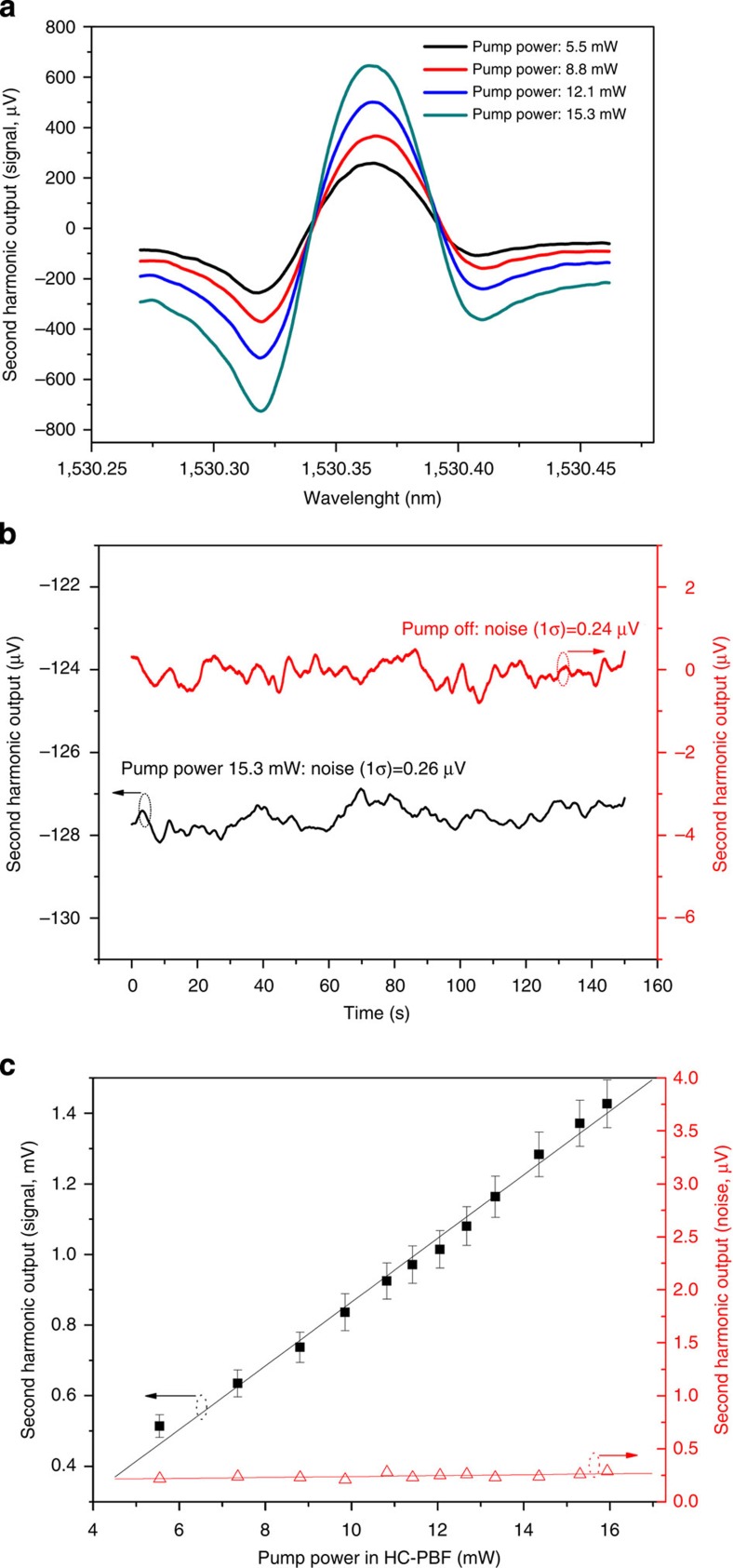
Experimental results for 10-m-long HC-PBF. (**a**) Second harmonic lock-in output (signal) when pump laser is tuned across the P(9) line of acetylene at 1,530.371 nm. (**b**) Second harmonic lock-in output when the pump wavelength is fixed to 1,530.53 nm. Black line: pump power is ∼15.3 mW. Red line: pump power is zero (off). The measurements were conducted consecutively for the two pump power levels but the results are displayed in the same panel for easy comparison. (**c**) Second harmonic signal and the s.d. of the noise as functions of pump power level. Error bars show the s.d. from five measurements and the magnitudes of the error bars are scaled up by 10-fold for clarity reason. The mean probe power level on PD2 is ∼50 μW. The gas concentration used is 10 p.p.m. acetylene balanced by nitrogen, and the experiments were conducted at room temperature. The amplitude of wavelength modulation was set to ∼2.2 times of the absorption linewidth. The time constant of the lock-in amplifier is 1 s with a filter slope of 18 dB Oct^−1^, corresponding to a detection bandwidth of 0.094 Hz.

The capability of HC-PBF-based PT spectroscopy for detecting weak absorbance was tested with the set-up shown in Fig. 2. The sensing HC-PBF sample is 10-m long and was fusion spliced to SMF-pigtails at both ends. Two microchannels were fabricated near the HC-PBF/SMF splicing joints for the purpose of pressurizing sample gas into the HC-PBF (see Methods). Figure 3a shows the second harmonic lock-in outputs for different pump power levels delivered to the HC-PBF when the pump wavelength was tuned across the P(9) line of acetylene in the ν_1_+ν_3_ band and the HC-PBF was filled with 10 p.p.m. acetylene balanced by nitrogen (N_2_). For the pump power of 15.3 mW, corresponding to a peak pump intensity of *I*_0_=40 kW cm^−2^, the peak-to-peak amplitude of the second harmonic signal is ∼1.37 mV. The second harmonic lock-in output when the pump is tuned away from the absorption peak to 1,530.53 nm is shown in Fig. 3b. The s.d. of the noise over 2-min duration is 0.26 μV, not much larger than the noise level (0.24 μV) when the pump is off. However, a constant bias exists when the pump is on, due to background absorption. The lower detection limit for a signal to noise ratio of unity is estimated to be 2 p.p.b. in NEC and 2.3 × 10^−9^ cm^−1^ in NEA, for a detection bandwidth of 0.094 Hz.

The second harmonic signal and the s.d. of the noise as functions of pump power level are shown in Fig. 3c. The signal amplitude increases approximately linearly with pump power while the noise level showed very little change. The 1*σ* NEC is better than 6 p.p.b. for pump power above 6 mW. This shows that p.p.b. level (acetylene) gas detection can be easily obtained with a relatively low power pump level, which is significantly better than the previously reported fibre-based LAS sensors.

It should be noted that the HC-PBF used in our experiment is not a true SMF^26^. It actually supports several higher-order modes and mode interference (MI) was identified as a major source of noise for the HC-PBF-based direct absorption sensors^6^. However, the MI was found to have minimal effect on the performance of the current HC-PBF-based PT sensor, evident from the very smooth trace of the second harmonic output as shown in Fig. 3a.

A closer examination of PT phase modulation and detection processes reveals that several factors would be responsible for the reduction of the MI. First, for the HC-PBF samples we used the cladding modes have already largely been removed. The first group of the higher-order mode (that is, LP_11_) still exists at the exit of the HC-PBF sample, but the power is relative small. For the 10-m-long sample, the power in the LP_11_ mode at the output end is estimated to be ∼0.03% of the fundamental mode. Second, the probe wavelength is fixed and the probe interferometer is servo-locked to the quadrature point. The residual high order mode of the probe beam, which interferences with the fundamental modes in the HC-PBF and the SMF in the reference arm, would only cause slow intensity fluctuation at the interferometer output. The fluctuation is small in amplitude and would translate into, via servo-control, a small shift of the operating point and should have minimal effect on the detection of the higher frequency PT-induced phase modulation. Third, the pump beam is filtered out before reaching the photodetectors, and more importantly the higher-order mode power of the pump would have minimal effect on the phase modulation of the fundamental mode. This is because the change of effective RI of a propagating core mode due to perturbation of core material (gas) RI is proportional to 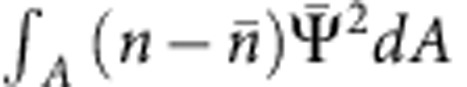, where *n* and 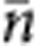 represent, respectively, the RI distribution for perturbed and unperturbed fibres, 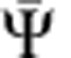 is mode field profile^27^. The integration is over the entire cross-section of the fibre. Since the induced RI change in gas is mainly due to density change, which tends to follow the intensity profile (heating profile) of the mode, the pump power in a higher-order mode tends to induce a RI perturbation that is ‘orthogonal' to the profile of the fundamental mode. More specifically, the LP_11_ mode has a two-lobe intensity profile, which results in a reduction of gas RI near the lobe intensity maxima and an increase of gas RI at the centre of the core. Such a RI perturbation would make the overlap integral with the fundamental mode approaching zero and hence have minimal effect on the effective RI of the fundamental mode. For the same reason, the effect of pump power in the fundamental mode would also have minimal effect on the effective RI of the higher-order mode. This means that the effective RI (and hence phase of the probe) of a mode is only significantly modulated by the pump power in the same mode, and the cross-modulation due to pump power in a different mode would be minimal. These and the fact that only a very small fraction of pump and probe power is in the higher-order mode suggest that the effect of the higher-order mode would have minimal effect on the performance of the HC-PBF-based PT spectroscopic sensors. However, further detailed modelling and experimental investigations are needed to quantify the influence of the higher-order mode, which could be a challenging task due to the complex fluid/thermal dynamic processes in gases and the surrounding fibre microstructure.

Environmental disturbance (for example, temperature and strain) affects the phase difference between the two interfering beams of MZI and hence the operating point. At laboratory environment, the disturbance is small, and, at low frequencies, our PZT-servo-loop worked well and the quadrature operating point is maintained well. The PT signal, which is at a much higher frequency (50 kHz), was found not significantly affected by the drift of the operating point. For field applications, the environmental disturbance could be unpredictable and the ability of maintaining quadrature operation for the current Mach-Zehnder scheme still needs to be tested. The fluctuation of the second harmonic output (see Fig. 3b) when the pump wavelength is tuned away from absorption peak was found being correlated with the output from PD1, which is used for MZI stabilization. This suggests that the active digital PZT servo is responsible for the noise level observed. The digital servo signal applied to the PZT introduces noise over a wide frequency range and the noise power within the system detection bandwidth is indistinguishable from the PT phase modulation signal, which sets the limit to concentration detection. The use of a passive phase demodulation scheme in a Sagnac loop configuration would improve the performance of the system^28^.

### Test of dynamic range

**Figure 4:**
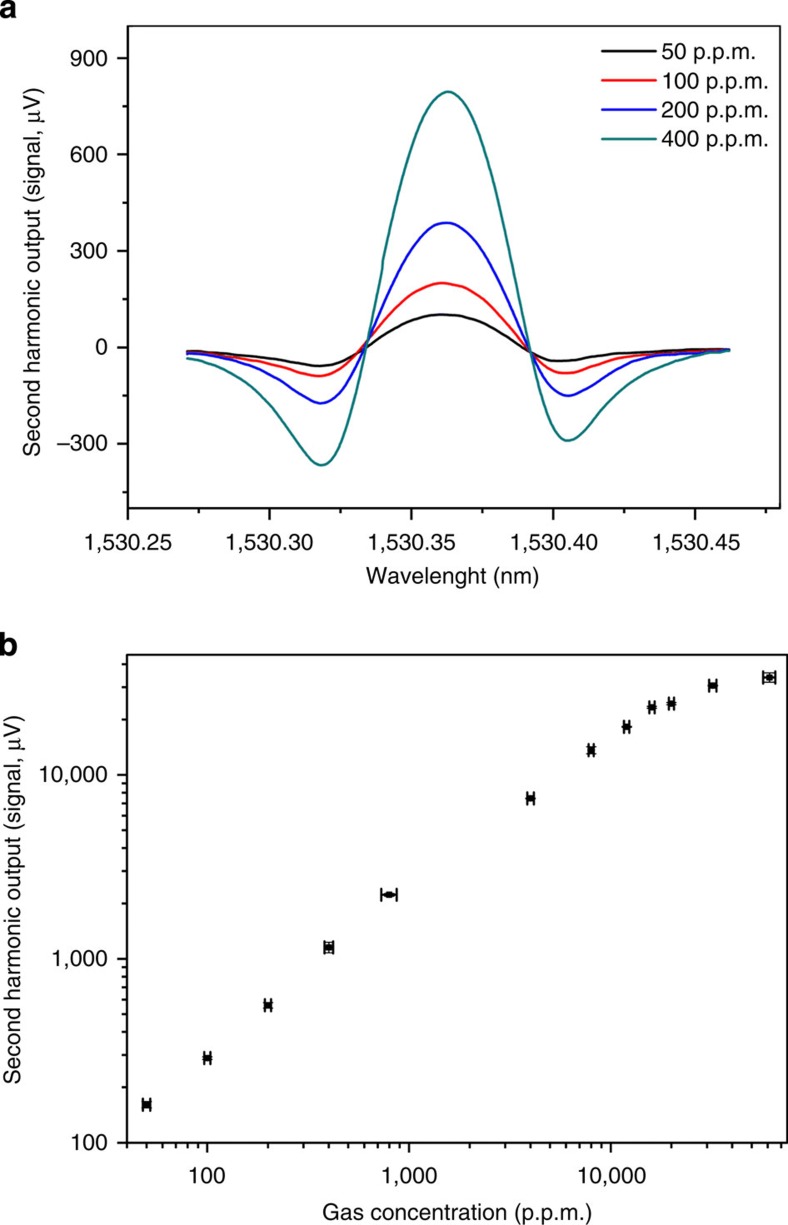
Second harmonic signal as function of gas concentration. (**a**) Second harmonic lock-in output signal when pump laser is tuned across the P(9) line of acetylene at 1,530.371 nm for 50, 100, 200, 400 p.p.m. acetylene concentration. (**b**) Second harmonic signal (peak-to-peak value) as function of gas concentration. Error bars in the horizontal axis are based on the accuracy of the two mass flow controllers we used to prepare different gas concentrations. Error bars in the vertical axis show the s.d. from five measurements and the magnitudes of the error bars are scaled up by 10-fold for clarity reason. The pump power in the hollow-core was estimated to be ∼25 mW and the mean probe power level on PD2 is ∼200 μW. The time constant of the lock-in amplifier is 1 s with a filter slope of 18 dB Oct^−1^, corresponding to a detection bandwidth of 0.094 Hz. The length of the sensing HC-PBF is 0.62 m.

The dynamic range of the HC-PBF-based PT spectroscopic system was tested with a 0.62-m-long HC-PBF. The experimental set-up used is slightly different from Fig. 2 (see Supplementary Fig. 2). The HC-PBF was spliced to SMF-pigtails while multiple microchannels were drilled into the hollow core of the HC-PBF (see Methods) for easy filling of the hollow core with different gas concentrations without the need for pressurization. Figure 4 shows the second harmonic lock-in output for acetylene concentration from ∼50 p.p.m. to ∼6% acetylene balanced with nitrogen. The lower acetylene concentrations (that is, 50 to 794 p.p.m.) were prepared by mixing pure nitrogen with 1% acetylene, while the higher concentrations were prepared by mixing nitrogen with 99.99% acetylene. An approximately linear relationship is obtained for acetylene concentration up to ∼1.6% and non-linearity starts to appear beyond this value. The lower detection limit in terms of 1*σ* NEC is estimated to be 30 p.p.b. (3.5 × 10^−8^ cm^−1^ in terms of NEA), giving a dynamic range of nearly six orders of magnitude (5.3 × 10^5^). Such a high performance has not been achieved with any fibre-based gas detection system reported previously.

The dynamic range of the PT sensor would be limited by the non-linear transfer function of the interferometer and the non-linear dependence of the phase modulation beyond the weak absorption approximation. For the experimentally used pump modulation frequency of 50 kHz, the scale factor k^*^, which is defined in equation (5), was determined to be 1.12 rad cm mW^−1^ m^−1^ (see Methods). For the results shown in Fig. 4, which used 25 mW pump power and 0.62-m-long HC-PBF sample, the phase shift at 1.6% acetylene concentration is calculated to be ∼0.233 rad, which is well within the 1% linear dynamic range of the interferometer transfer function (that is, Δ*φ*≤0.1*π*). However, the pump power is absorbed significantly and reduced to ∼30% at the output of the HC-PBF sample, suggesting that the weak absorption assumption is no longer valid and the heat generation rate should follow an exponential function of the absorbance which is the factor responsible for the non-linear behaviour for larger acetylene concentrations.

### Shot-noise-limited performance

**Figure 5:**
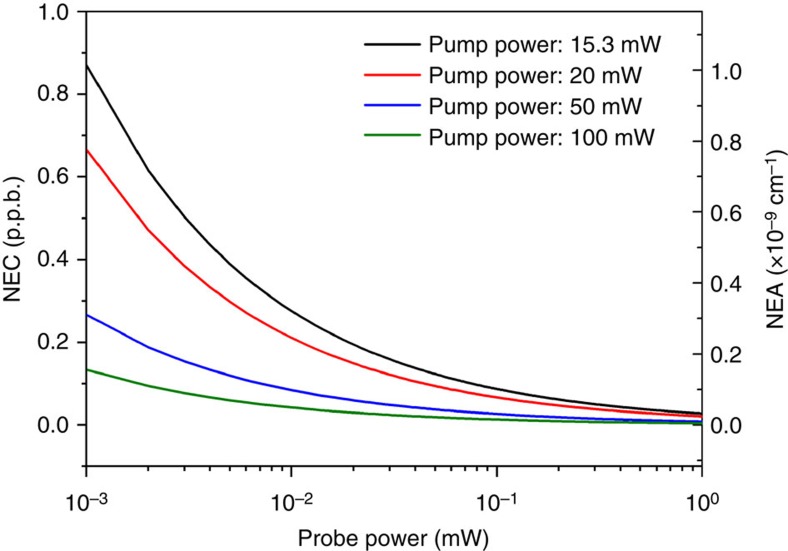
Shot-noise limit in terms of NEC and NEA for different pump and probe power levels. The pump wavelength is tuned to the P(9) absorption line of acetylene at 1,530.371 nm and the length of HC-PBF is 10 m. Detection bandwidth is 0.094 Hz, corresponding to 1 s time constant of the lock-in with a filter slope of 18 dB Oct^−1^. Probe wavelength *λ*_probe_=1,556.59 nm, *v*_probe_=*c/λ*_probe_. Detector quantum efficiency *η=*0.8, Planck constant *h*=6.626 × 10^−34^ J s.

The lower detection limit due to shot-noise may be estimated by^15^


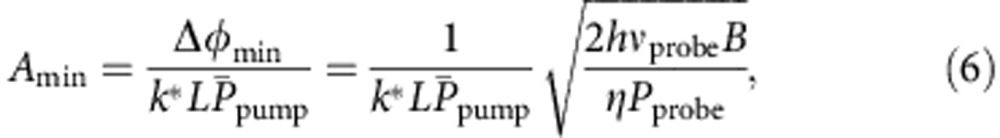


where *h* is the Planck constant, *B* the detection bandwidth and *η* the efficiency of the photodetector. *v*_probe_ and *P*_probe_ are, respectively, the frequency and power of the probe beam. With equation (6) and the parameters used in our experiments, the shot-noise-limited detection sensitivity was calculated and shown in Fig. 5. For the 15.3 mW pump power, 50 μW probe power, 10-m-long HC-PBF, and a detection bandwidth of 0.094 Hz, the shot-noise-limited NEC and NEA are, respectively, 120 p.p.t. acetylene and 1.4 × 10^−10^ cm^−1^. It should be mentioned that the above shot-noise calculation did not consider the effect of wavelength modulation. In our experiment, we used wavelength modulation and second harmonic detection, which would deteriorate the noise limit by a factor^29^ of ∼4 and give an estimated detection limit of 480 p.p.t. NEC or 5.6 × 10^−10^ cm^−1^ NEA. The calculated shot-noise-limited NEC is ∼4 times better than the experimentally obtained detection limit.

## Discussion

In summary, we have developed a novel all-fibre optical sensing technique based on PT phase modulation in a HC-PBF, which will enable high precision cost-effective fibre-optic sensors for real-time trace chemical detection. Preliminary experiments with acetylene gas demonstrated p.p.b. level detection limit and an extremely large linear dynamic range of nearly six orders of magnitude.

For comparison, we list in Table 1 the lower detection limits of some previously reported direct absorption fibre-optic gas sensors. Although the results were obtained with different gases, system configurations and parameters^8^,^30^,^31^,^32^,^33^,^34^, we may, however, compare the performance of these sensors in terms of NEA. With 5.1-m-long absorption path, the HC-PBF-based sensor demonstrated a NEA of 1.6 × 10^−6^ cm^−1^, which is ∼700 times worse than the performance of our current HC-PBF PT sensor.

With similar parameters as used in the PT sensor, we calculated the shot-noise limit of a direct absorption sensor for 50 μW optical power reaching the photodetector and found to be 20 p.p.t. in terms of NEC or 2.3 × 10^−11^ cm^−1^ in NEA. Again, the detection limit would be worsening by a factor of 4 if wavelength modulation/second harmonic detection is used^29^. These values are ∼6 times smaller than the shot-noise limit of our PT sensor with 15.3 mW pump power, showing that the performances of current direct absorption optical fibre sensors are far away from their shot-noise limits. Current commercial HC-PBFs are not true SMFs^26^, and MI is a major factor that limits the performance of the HC-PBF-based direct absorption sensors^6^. However, MI has negligible effect on the performance of our HC-PBF-based PT spectroscopic sensors.

Similar to tunable diode LAS, the diode laser-based PT spectroscopic sensor could be made self-calibrating against intensity changes in the pump and probe. Assuming the interferometer is maintained at quadrature, the intensity modulation at the interferometer output would be linearly proportional to the absorption-induced phase change, that is,


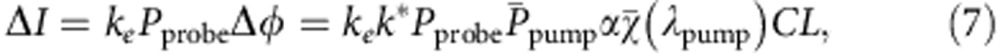


Where *k*_*e*_ is a constant and *k*_*e*_*P*_probe_ is the proportionality factor related to interferometric detection and 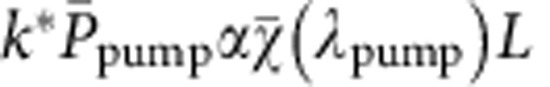 is a factor related to the PT phase modulation. *k*_*e*_*P*_probe_ could in principle be self-calibrated by for example normalizing it against the DC component or peak-to-peak magnitude of the interferometer output. 
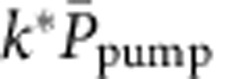
 could also be calibrated by detecting the residual intensity modulation of pump, similar to that for the direct absorption sensors^35^. The combination of the two would allow self-calibration of the PT HC-PBF sensor against both pump and probe power variations.

Compared with free-space PT interferometry systems, the use of HC-PBF offers significant benefits. First, the pump, probe and sample overlap perfectly within the hollow core, and the efficiency of sample excitation and probing is extremely high. Second, the MFDs of the fundamental mode of the state-of-the-art commercial HC-PBFs are as small as 5–10 μm (refs 18, 19, 20), over two orders of magnitude smaller than the diameter of a typical free-space beam (1–2 mm)^13^,^14^,^15^,^16^,^17^. This means that for the same power level, the pump intensity in a HC-PBF can be over four orders of magnitude higher, which significantly enhances the heat production. In contrast, to achieve the same pump intensity, the pump laser power required is much less, allowing the use of a lower cost pump source. Third, the fundamental mode in a HC-PBF is well confined within the hollow core with low loss over a distance of metres to kilometres, and the absorption distance can then be 10 to 10^4^ times longer than a free-space system. The reduced beam size and increased absorption distance resulted from using HC-PBF would possibly enhance the overall efficiency of the PT phase modulation by 5 to 8 orders of magnitude, which would enable highly efficient PT fibre-optic devices. However, the thermal/fluid dynamic processes in a gas-filled HC-PBF could be very complex and significantly different from a free-space system; the efficiency of PT modulation would also depend on the modulation frequency. Further work is needed to understand the various processes involved and evaluate the full potential of the HC-PBF-based PT interferometric devices.

Current HC-PBFs are primarily operating in the visible and NIR where molecular absorptions of most gases and analytes are weaker than that in the MIR. However, the overall significantly enhanced efficiency as discussed above would compensate the signal reduction due to the weaker absorption. Operating in the NIR also allows the use of cheaper and more compact photonic components compatible with standard telecom optical fibres, making it possible to develop cost-effective all-fibre phase modulation devices, and remote, multiplexed and distributed sensing systems.

HC-PBF-based PT spectroscopy can readily be applied to detect other types of gases. The HC-1550-02 fibre we used in this investigation has a transmission window from 1,500 to 1,700 nm, which covers a range of gases such as CO_2_, CO, CH_4_, N_2_O, H_2_S, NH_3_, HI, C_4_H_6_ and could then be used to detect these gases. Furthermore, there are a range of HC-PBFs with transmission bands from 400 nm to 2 μm available from NKT Photonics, allowing a wider range of gas species to be detected.

The extension of HC-PBF-based PT spectroscopy to liquid analysis is straightforward. When combined with fibre-based microfluidic arrangement^34^,^36^, the technique would provide an ultra-sensitive means for environmental, chemical and bio-chemical detection with micro/nano litre sample consumption. The absorption-induced PT phase modulation in HC-PBF would allow novel all-fibre photonic devices, fluid sensing networks^5^ and distributed sensing system based on detecting phase modulation along the entire length of HC-PBF with, for example, coherent phase optical-time-domain-reflectometry^37^. The work could also be extended to other microstructured fibres such as suspended-core and Kagome fibres and to other wavelength regions from ultra-violet to MIR^34^,^38^. The ability for transmitting high-intensity laser beams within HC-PBFs could also be exploited for ultra-sensitive nonlinear fibre-optic PT spectroscopy^39^.

## Methods

### Preparation of HC-PBF samples

The fibre samples were prepared by splicing HC-1550-02 fibres to SMFs with the Ericsson FSU975 fusion splicer. The fusion duration was set to 0.2 s, and the offset and overlap were 45 and 10 μm, respectively.

For the 0.62-m sample, the output end of the HC-1550-02 was fusion spliced to SMF with low fusion current to prevent the collapse of air-holes^40^. The input end was, however, fusion spliced to SMF with a larger fusion current of ∼16 mA. This collapses the cladding air-holes to some extent and helps to minimize the excitation of the cladding modes and hence reduces modal interference (see Supplementary Fig. 3 and Supplementary Discussion). Fifteen microchannels were drilled along the sample by use of an 800-nm femtosecond laser^6^, and the spacing between the microchannels ranges from 2 to 7 cm. The average loss over 1,500 to 1,600 nm was estimated to be ∼0.02 dB per channel, and the total loss from the input to output SMF is ∼4 dB. The drilling of microchannels was found not significantly increasing the MI (see Supplementary Fig. 4 and Supplementary Discussion).

The diameter of the microchannels is ∼5 μm, as shown in the SEM image in the Supplementary Fig. 4. At atmospheric pressure, the Knudsen number of the target gas is much smaller than 1, as the mean free path of the gas molecule is much smaller than the diameter of the hollow core. The diffusion process is close to the continuum state and the microchannels with depth of ∼60 μm do not affect the speed of gas diffusion significantly.

For the 10-m sample, the HC-PBF was fusion spliced to SMFs at both ends with a fusion current of 12.5 mA. Compared with the 0.62-m sample, the cladding modes are almost completely attenuated and the magnitudes of higher-order core modes are also reduced considerably (∼0.03% of the fundamental mode power) because of the relatively longer length of HC-PBF used. Two microchannels were drilled from aside of the HC-PBF near the HC-PBF/SMF splicing joints and the total loss from the input to output SMF is ∼4 dB.

### Gas detection with the 10-m-long HC-PBF

The experimental setp-up used for the 10-m-long HC-PBF sample is shown in Fig. 2. The sections of the 10-m-long HC-PBF with microchannels were placed within two 10-cm-long tubes with diameter of 1 cm. One of the tubes was connected to a high-pressure gas cylinder containing 10 p.p.m. acetylene balanced by nitrogen while the other left open to atmosphere. The HC-PBF was first filled with the 10 p.p.m. acetylene with ∼2.5 bar pressure for ∼2 h, and then depressurized and sealed before taking measurements. The experiment was conducted at room temperature.

The pump power delivered to the HC-PBF was estimated by detecting pump power at the output of Filter 2, deducting the losses of the circulator and the SMF/HC-PBF splice.

To estimate the noise level, the second harmonic lock-in output when the pump was tuned to 1,530.53 nm (away from acetylene absorption peaks) was sampled at the rate of 40 samples per second and recorded, and the s.d. of the noise calculated.

### Gas detection with the 0.62-m-long HC-PBF

The 0.62-m-long HC-PBF was placed inside a 23 cm × 15 cm × 7 cm gas chamber, and the sample gas with different concentrations was prepared by mixing 1 or 99.99% acetylene with pure nitrogen at different flow-rate ratios at atmospheric pressure through two mass flow controllers. For preparing the 50, 100, 200, 400, 794 p.p.m. acetylene samples, the flow rates used are, respectively, 1, 2, 4, 5 and 5 sccm (standard cubic centimetre per minute) of 1% acetylene with 199, 198, 196, 120 and 58 sccm of pure nitrogen. For preparing the 0.4, 0.8, 1.2, 1.6, 2, 3.205 and 6.098% gas samples, the flow rates used are, respectively, 1, 2, 3, 4, 5, 5 and 5 sccm of 99.99% acetylene with 249, 248, 247, 246, 245, 151 and 77 sccm of pure nitrogen. The sample gas diffuses into the hollow core through the microchannels made on the HC-PBF.

The experimental set-up used for the 0.62-m-long HC-PBF sample is similar to the one shown in Fig. 2, but the pump power is injected into the HC-PBF via one port of FC2 (see Supplementary Fig. 2). The methods used for calculating the pump power level to the HC-PBF and the s.d. of the noise are similar to the 10-m sample.

### Determination of scale factor k*

The 0.62-m-long HC-PBF sample was filled with 1% acetylene, and the pump power input to the HC-PBF was estimated to be 35 mW. The amplitude of wavelength modulation was set to ∼3.2 times of the absorption linewidth and the centre wavelength of the pump was tuned to 1,530.371 nm. The interference fringe contrast was estimated by observing the PD2 output with an oscilloscope as illustrated in Supplementary Fig. 3, and the maximum and minimum voltages without phase stabilization were first measured to be 0.2 and 2.9 V. The peak-to-peak modulation signal from PD2 when the interferometer is stabilized at quadrature was determined to be 0.195 V. By comparing this value with the fringe contrast, the amplitude of the induced phase modulation was determined to be 0.145 rad. Since the absorption of 1% acetylene is not weak, we calculated the average pump power by integrating the absorption-induced power variation over the entire length of the HC-PBF and obtained k*α=1.3 × 10^−6^ rad p.p.m.^−1^ mW^−1^ m^−1^ or k^*^=1.12 rad cm mW^−1 ^m^−1^.

## Author contributions

W.J. proposed and coordinated the work. Y.C. jointly developed the idea, built the experimental set-up and carried out preliminary experiments. F.Y. and H.L.H. modified the set-up. F.Y. prepared the samples, conducted gas detection measurements and achieved the results reported. W.J. and F.Y. analysed the mode interference noise. We performed data analysis and drafted the manuscript together.

## Additional information

**How to cite this article:** Jin, W. *et al*. Ultra-sensitive all-fibre photothermal spectroscopy with large dynamic range. *Nat. Commun*. 6:6767 doi: 10.1038/ncomms7767 (2015).

## Supplementary Material

Supplementary Information Supplementary Figures 1-4, Supplementary Discussion and Supplementary References

## Figures and Tables

**Table 1:** Detection limits of absorption-based optical fibre gas sensors.

Gas type	Wavelength (μm)	Gas cell	Technique	Integration time (s)	NEC (p.p.m.)	NEA (cm^−1^)
Methane^30^	1.6659	5 cm open path	WMS	0.64	500	5.6 × 10^−5^*
Methane^31^	1.666	13.7 cm HC-PBF	WMS	Not stated	158	1.8 × 10^−5^*
Methane^8^	1.645	5.1 m HC-PBF	DAS	Not stated	10	1.6 × 10^−6^
Acetylene^32^	1.52	1 m HC-PBF reference cell, 14 cm measurement cell	CS	4	100	1.3 × 10^−4^*
Acetylene^33^	1.53037	27 m HC-PBF	DAS	Not stated	50	5.8 × 10^−5^*

CS, correlation spectroscopy; DAS, direct absorption spectroscopy; WMS, wavelength modulation spectroscopy.,

^*^NEA data were calculated from NEC data and the absorption line strength from HITRAN database^24^.
