# Supplementary material for: Ultra-sensitive all-fibre photothermal spectroscopy with large dynamic range
Source: Nat Commun. 2015 Apr 13;6:6767. doi: 10.1038/ncomms7767 (PMC4403440; doi:10.1038/ncomms7767)
Supplement: Supplementary Information — Supplementary Figures 1-4, Supplementary Discussion and Supplementary References [file ncomms7767-s1.pdf]

Supplementary Figures

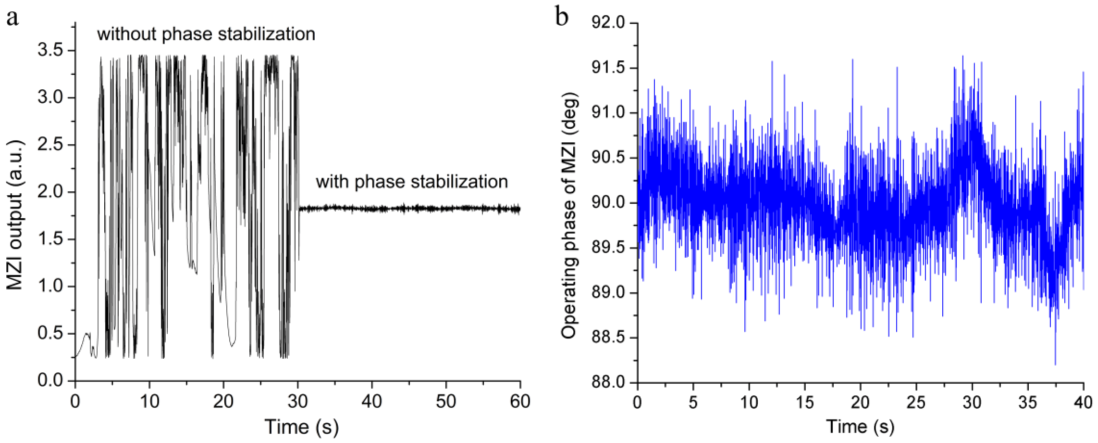

Supplementary Figure 1: Mach-Zehnder interferometer (MZI) phase stabilization. (a) DC output of the MZI with and without phase stabilization. **(b)** Performance of MZI stabilization in terms of phase fluctuation around the quadrature point. The sampling frequency is 40 samples per second. The MZI stabilization was achieved with a similar approach as reported in [1]. The output varies randomly between the maximum and minimum without phase stabilization and reaches an approximately constant value after the phase stabilization is applied. The performance of the MZI stabilization in terms of phase fluctuation from the target quadrature point was calculated by comparing the output intensity fluctuation of the stabilized MZI with the interference fringe contrast (i.e., from ~0.2 to ~3.4 as seen from **a**). The results are shown in **b**. The standard deviation (s.d.) of the phase fluctuation is calculated to be 0.55 °.

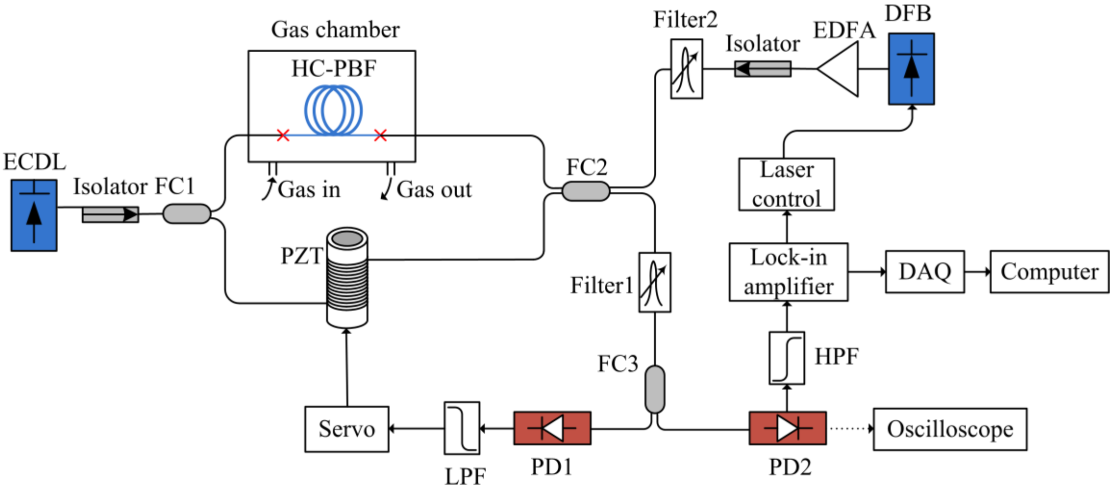

Supplementary Figure 2: Experimental setup for gas detection with 0.62-meter-long HC-PBF. ECDL, external-cavity diode laser (the probe); DFB, distributed feedback laser (the pump); FC, fibre coupler; PZT, piezoelectric transducer; PD, photodetector; LPF, low-pass filter; HPF, high-pass filter; EDFA, erbium-doped fibre amplifier; DAQ, data acquisition. The splitting ratios of FC1 and FC2 are respectively 70/30 and 50/50. The splitting ratio of FC3 is 90/10. Filter 1 is used to filter out the residual pump and Filter 2 to minimize the effect of EDFA's ASE noise. The pump power in the hollow-core is estimated to be ~21 mW and the mean probe power level on PD2 is ~300 μW.

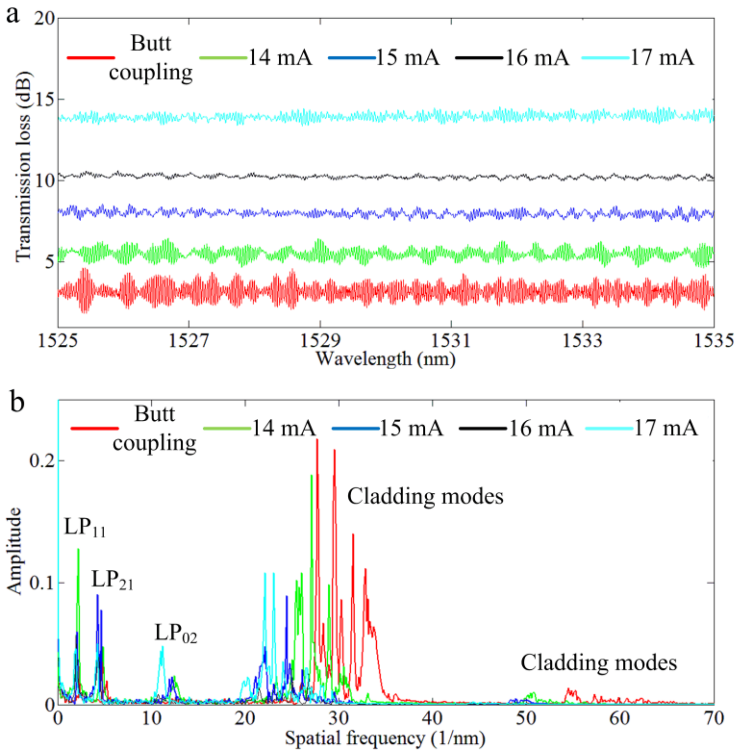

Supplementary Figure 3: Transmission measurement of 0.6-m-long HC-1550-02 fibre sample. (a) Measured (normalized) transmission spectrums with different fusion currents from 14 to17 mA applied to the input splice; for improved clarity, 2, 4, 6, and 8 dB offsets were intentionally introduced respectively to the 14, 15, 16 and 17 mA curves; **(b)** the Fourier transform of the transmission spectrums in **a**. Light from a tunable external-cavity diode-laser source was launched into the 0.6-m-long HC-PBF sample through a SMF pigtail and the transmission spectrum was measured with a photodetector. The laser source and photodetctor are parts of an Agilent 81910A Photonic All-Parameter Analyzer. The wavelength tuning range of the laser was set from 1,525 to 1,535 nm and the tuning resolution was 0.001 nm. The 0.6-m-long HC-1550-02 fibre was fusion spliced to SMFs at both ends by use of an Ericsson FSU-975 fusion splicer. For the splice at the output end of the HC-PBF, the fusion current used was low with short duration to prevent collapse of air-holes [2]. The input end of the HC-1550-02 fibre was however spliced to SMF with different fusion currents from 14 to 17 mA. The fusion duration was set to 0.2 s, and the offset and overlap were 45 and 10 μm, respectively. The spectrums were normalized to the source spectrum measured directly from its SMF pigtail. The higher frequency oscillations were significantly reduced for the splice with fusion current of 16 mA. The different peaks in **b** correspond to interferences of the fundamental core mode with different higher order core and cladding modes, which are labeled in **b**.

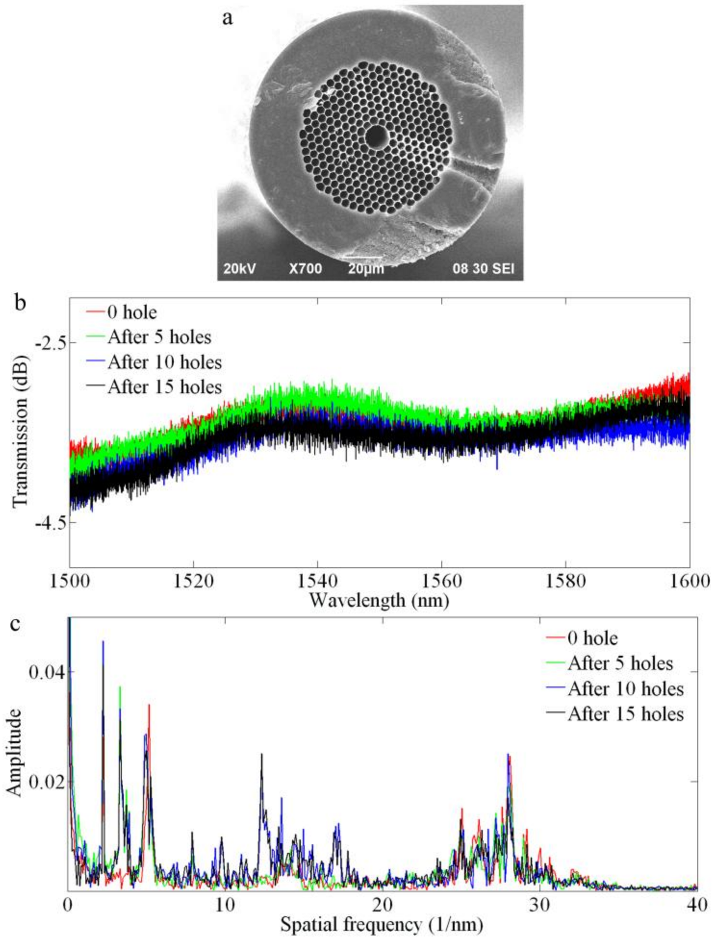

Supplementary Figure 4: Transmission measurement of 0.62-m-long HC-1550-02 fibre sample with different number of micro-channels. (a) SEM images of HC-1550-02 fibre with a typical micro-channel. (**b)** Transmission spectrums of a 0.62-m-long HC-PBF with different number of micro-channels (side-holes). (**c)** The Fourier transforms of the transmission spectrums in **b**. A 0.62-m-long HC-1550-02 fibre was fusion spliced to SMF at both ends and the fusion parameters for the input splice were optimized to minimize the MI described before. A femtosecond laser was used for to fabricate the micro-channels, as described in [3]. The transmission loss was monitored in situ with an optical spectrum analyzer in combination with a broadband light source. The transmission spectrums were recorded by an optical spectrum analyzer with 0.01 nm wavelength resolution. The average loss from 1,500 to 1,600 nm for 15 side-holes was measured to be ~0.2 dB, giving an average loss of less than 0.02 dB per channel. The magnitude and the spectral contents of the MI showed no significant increase for 5 to 15 side-holes, as can be seen in **b** and **c**.

Supplementary Discussion

Mach-Zehnder interferometer (MZI) stabilization

The phase fluctuation as shown in Supplementary Figure 1b is typically at a lower frequency as compared with the photothermal (PT) phase modulation (50 kHz in our experiments) and it would not directly affect the lower detection limit of gas concentration and only affect the phase-to-intensity conversion coefficient (i.e., the slope of the phase to intensity transfer function) slightly. For the phase error of 0.55 ° away from quadrature, the slope change would be $1-\cos(0.55^{\circ})=4.6\times10^{-5}$. For an applied gas concentration of *C*, the relative measurement uncertainty due to this slope variation would be $\Delta C/C=4.6\times10^{-5}$. In our experiment of evaluating the lower detection limit, we used 10 ppm of acetylene and the uncertainty is then estimated to be $4.6\times10^{-5}\times10$ ppm = 0.46 ppb, ~4 times smaller than the estimated lower detection limit of 2 ppb. However, since our servo-loop is digitally controlled by a computer, and digital signal applied to the PZT generates sharp transient peaks, which results in higher frequency noise and affect the limit for gas detection.

Mode interference (MI) reduction

The HC-1550-02 fibre used in our experiments is not a true single mode fibre, it supports several higher-order core and cladding modes. Mode interference (MI) in HC-PBF has been identified to cause fluctuation of background, which affects the stability and noise performance of gas sensors [3, 4]. We have studied MI occurring in short (<3 m) HC-PBF spliced to standard SMFs at both ends and demonstrated that MI can be substantially reduced by optimizing the fusion current used to splice the HC-PBF with SMFs.

The spatial frequencies of the peaks shown in Supplementary Figure 3b, which represent different MI components, reduce for increasing fusion currents from 14 to 17 mA. This is because that we used the same HC-PBF sample for all the experiments and the HC-PBF sample was cleaved and became shorter after each measurement. The HC-PBF was ~0.6 m long for 14 mA and slightly shorter for 17 mA. The rate of decrease in the magnitude of the MI due to cladding modes is much faster than the core modes for fusion current from 14 to 16 mA. When the fusion current was increased from 16 to 17 mA, the insertion loss become significantly higher and the MI due to LP_{02} mode increased as well. The s.d. of the MI noise for the 16 mA fusion current is ~6 times smaller than direct butt-coupling and only ~1 dB additional loss is introduced in the wavelength range of 1,525-1,535 nm. The MI reduction was demonstrated for several samples with lengths below 3 m, and the magnitudes of MI due to cladding mode can be significantly reduced by proper selection of fusion

current.

With increasing fusion current from 14 to 16 mA, an increasingly larger degree air-hole collapse in the cladding was observed. For 16 mA, all the cladding holes would collapse over a short section of the HC-PBF, which would induce large loss to the cladding modes. However, the hollow-core remains relatively unaffected, and hence the core modes are relatively unaffected. The short collapse region would act as a mode filter to reduce the power of the cladding modes but not the core-modes. When the fusion current was increased to 17 mA, the hollow-core was also affected, which results in a larger transmission loss to the fundamental core mode as well as excitation of the higher-order LP_{02} core-modes.

Consequently, for the 0.62-m-long HC-PBF sample that was employed in our experiments, we used 16 mA fusion current for the input splice.

Supplementary References

[1] Rosenthal, A., Kellnberger, S., Sergiadis, G. & Ntziachristos, V. Wideband fiber-interferometer stabilization with variable phase. *IEEE Photon. Technol. Lett.* **24,** 1499-1501 (2012).

[2] Xiao, L., Demokan, M. S., Jin, W., Wang, Y. & Zhao, C.-L. Fusion splicing photonic crystal fibers and conventional single-mode fibers: microhole collapse effect. *J. Lightwave Technol.* **25,** 3563-3574 (2007).

[3] Jin, W., Ho, H. L., Cao, Y. C., Ju, J. & Qi, L. F. Gas detection with micro- and nano-engineered optical fibers. *Opt. Fiber Technol.* **19,** 741-759 (2013).

[4] Parry, J. P. *et al.* Towards practical gas sensing with micro-structured fibres. *Meas. Sci. Technol.* **20,** 075301 (2009).
